# Toward Universal Health Coverage: Regional Inequalities and Potential Solutions for Alleviating Catastrophic Health Expenditure in the Post-poverty Elimination Era of China

**DOI:** 10.34172/ijhpm.2023.7332

**Published:** 2023-02-20

**Authors:** Yanan Luo, Zhenyu Shi, Dan Guo, Ping He

**Affiliations:** ^1^Department of Global Health, School of Public Health, Peking University, Beijing, China.; ^2^School of Public Health, Peking University, Beijing, China.; ^3^China Center for Health Development Studies, Peking University, Beijing, China.

**Keywords:** Universal Health Coverage, Regional Inequalities, Catastrophic Health Expenditure

## Abstract

**Background:** This study took Beijing as an example to estimate the incidence and regional inequalities of catastrophic health expenditures (CHE) in a megacity of China.

**Methods:** This study used data from the Health Services Survey Beijing (HSSB) 2018. Logistic regressions were used to investigate the risk factors for experiencing CHE, and concentration curves, the concentration index and its decomposition method based on probit models were used to estimate the inequalities in CHE.

**Results:** CHE occurred in 25.51% of the households of the outer suburb villages, 6.78% of the households of the inner-city area communities, 17.10% of the households of the villages of the inner-city areas, and 11.91% of the households of the communities of the outer suburbs. In areas in the outer suburbs, households with private insurance coverage were associated with a lowered risk of CHE, and lower educational attainment and lower occupational class were related to an increasing risk of CHE. This study also discovered pro-rich financing disparities in CHE in Beijing, with the outer suburbs having the highest levels of CHE disparity. When it comes to the observed contributions of disparities in CHE, a significant portion of them is connected to the sorts of occupations, educational levels, and residential status.

**Conclusion:** The impoverishment brought on by medical expenses and CHE must still be taken into account in the post-poverty elimination era. The megacity of China was discovered to have significant regional differences in the incidence of pro-rich financing inequity in CHE. Disparities in socioeconomic status (SES), one of the controllable variables, may be a key area to address to lower the risk and minimize CHE inequality in megacities towards the path to UHC. Additionally, it is important to consider the financial protection impact of inclusive supplementary medical insurance on lowering the likelihood of CHE in the periphery areas.

## Introduction

**Key Messages**

** Implications for policy makers**

- In the post-poverty elimination era, the impoverishment from medical expenses and catastrophic health expenditures (CHEs) still needs to be noted. Tackling the socioeconomic inequalities of health systems should be considered to achieve universal health coverage (UHC).
- Reducing the occurrence of CHE and its inequality has promising prospects in the megacity of China. More concerns need to be diverted into the reduction of disease economic burden in households from both “urban villages” and villages in the outskirts of megacities.
- The modifiable determinants, socioeconomic status (SES), may be one of the effective interventions to reduce the risk and narrow the inequality of CHE in megacities.
- Policy development, such as targeting supplementary medical insurance in developing regions, needs to be the primary focus.

** Implications for the public**

Publics are encouraged to increase their socioeconomic status (SES), which may be one of the main effective ways to reduce both the risk and inequality of catastrophic health expenditure (CHE) in the post-poverty elimination era. SES is regarded as an important modifiable social determinant of health, and tackling modifiable social determinants is an essential way to improve health equity. Financial protection from inclusive supplementary medical insurance, such as the Beijing Inclusive Medical Insurance Program, in which healthy applicants and individuals with preexisting conditions can be insured and have access to claims with a low premium rate but are more insurable, may be of great help to be out of pocket reduction, particularly in vulnerable areas.

China has made great strides in health insurance coverage since a new round of health system reforms in 2009. However, a remarkable discrepancy still exists between such coverage and World Health Organization (WHO) standards of universal health coverage (UHC). UHC requires the breadth (population), depth (range of service) and height (extent to which health service costs are covered) of coverage,^1^ while despite the population coverage increasing in China, the height and depth still remain at a low benefit level.^2,3^ At the end of 2020, over 98 million impoverished people in rural areas lifted out of poverty in China, but the problems of poverty-returning due to diseases are still prominent. Both the weak control for health expenditures and unequal geographical distribution of healthcare services may fall short of protecting people from catastrophic health expenditures (CHEs).^4^ In the post-poverty elimination era, detecting the potential solutions of protecting CHE and its regional inequalities are important issues, which can help to consolidate poverty elimination achievements and promote the equality of opportunities and better access to health services.

 Megacities are the areas that are the closest to achieving the goals of UHC. As a consequence of rapid urbanization, megacities, along with economic booming and highly aggregated quality public resources, bring many health benefits to dwellers.^5^ However, the problems of imbalanced development and uneven distribution of health resources are more prominent, impeding equitable access to demanded health services and UHC for dwellers. Beijing is one of the largest megacities in China with the highest quality but the most prominent unbalanced medical resource distribution. In 2020, the number of hospital beds (per 1000 people) reached only 5.8 (<the nation average of 6.3) and was lower than that of other international megacities (Tokyo = 9.6, Paris = 12). Nevertheless, 13.9 hospital beds per 1000 people in urban cores of Beijing, which was far above that in peripheral areas.^6^ In Beijing, over 70% of hospitals are located in inner city areas, and most high-quality health resources are highly aggregated in urban cores,^7,8^ leading the impoverished and suburban area residents to high exposure to poverty-returning due to diseases. Therefore, it is an urgent issue in megacities to analyze the regional inequalities of impoverishment in terms of medical expenses and CHE.

 Although a series of studies have detected the determinants of CHE and its individual level inequalities, very few of them pay attention to inequalities at the regional level, and none of them focus on this issue in the setting of megacity areas. Here, we took Beijing as an example to identify the regional inequalities of CHE in megacities and to decompose the contributors, which could provide guidance for policy-makers in alleviating the weak health expense control and unequal access to health services in megacities and promoting the achievement of UHC in the post-poverty elimination era.

###  Background of Health Insurance Schemes in China

 The health insurance schemes in China engage that “fully covered by public health insurance, and private health insurance as supplements to the public schemes.” In the past, there were four main classes of health insurance schemes in Beijing, China: (1) urban employee basic medical insurance (UEBMI), which is mandatory for urban residents in employment and is paid for with employer and employee contributions; (2) the new cooperative medical scheme (NCMS) established in 2003, which is a voluntary insurance scheme for rural residents; (3) urban resident basic medical insurance (URBMI), covering urban residents without formal employment; and (4) private health insurance with funding by individuals, which is used by Chinese residents to supplement the government mandated public health schemes and foreigners in China.^9^

 However, complaints for unaffordable basic health services, medical impoverishment due to high out-of-pocket health expenditures, and increasing health disparities gradually increased. In response to this concern, the government has started an ambitious new round of healthcare-system reform since April 2009, with the goals of achieving the universal coverage of essential health services for all citizens by 2020, which made great difference in the expansion of health insurance, public hospital reform and the strengthening of primary care.^10,11^ The proportion of the Chinese population covered by public health insurance reached 95% in 2011.^12^

 Although NCMS and URBMI operated smoothly and laid the foundation for China to approach UHC, with the increasing population migration, some migrants were covered by both NCMS and URBMI, which led to financial burdens on individuals and public finance. To avoid these overlap coverages, improve the equity and advance the aims of UHC, the “Opinion on the integration of basic medical insurance systems between urban and rural residents” was issued in 2016, which required that NCMS and URBMI should be integrated into a new urban–rural resident medical insurance (URRMI) system.^13^ In 2018, Beijing completed the integration of URBMI and NCMS into URRMI scheme programs. All urban residents without formal employment and rural residents are eligible for the URRMI.

 The reimbursement ratio and the deductible schemes for public health insurance vary based on the location across China. In Beijing, regarding UEBMI, for outpatient services, the deductible is CNY (Chinese Yuan) 1300-1800/year, and the reimbursement ratio is 90% with an out-of-pocket limit of CNY 20 000/year; for inpatient services, the deductible is CNY 650-1300/year, and the reimbursement ratio is 85%-99.1% with an out-of-pocket limit of CNY 500 000/year. The deductible for URRMI is CNY 100-500/year for outpatient services with a reimbursement ratio from 50%-55% and CNY 150-1300/year for inpatient services with a reimbursement ratio from 75%-80%, which depend on age and areas of cover. The out-of-pocket limit is CNY 4500/year for outpatient services and CNY 250 000/year for inpatient services.

 However, even with public health insurance, there are some copay requirements at the point of accessing some care, and the out-of-pocket rates associated with public health insurance still remain a major financial challenge to patients with severe illness. As such, the government proposed establishing a multilevel health insurance system in which public health insurance is to secure basic healthcare, and private health insurance is to be a supplement focusing on high-cost healthcare.^12^ In Beijing, it launched the universal private health insurance program “Beijing Inclusive Medical Insurance Program (*Beijing Huimin Jiankangbao* in Chinese),” which is available to all residents in Beijing. The policyholders can receive reimbursements of up to CNY 3 million for medical expenses included or not covered by the medical insurance reimbursement list with only paid for CNY 195/year.

## Methods

###  Data

 Data from the Health Services Survey Beijing (HSSB) in 2018 were used in this study. HSSB aims to collect information on socioeconomic conditions, health status, health insurance enrollment, health service demands and utilization and medical expenditures among residents by face-to-face interviews with structured questionnaires, which is one of the important parts of the National Health Services Survey. This survey was organized by the National Health Commission of the People’s Republic of China and was conducted every 5 years from 1993 to 2018. Compared with waves from 1993 to 2013, HSSB 2018 is the first to involve all districts/counties of Beijing in the sampling units, which has favorable regional representation. By using a multistage, stratified, probability proportional to size sampling method, 16 districts/counties, 205 communities/villages, 12 303 households, and 29 197 individuals in Beijing were randomly selected. More details about the methodology can be found elsewhere.^14^ This study excluded individuals without information on types of residential areas, income, gender, educational attainment, occupation, smoking, drinking and medical insurance and included 25 297 participants in our final analysis.

###  Availability of Data and Materials

 The datasets of the HSSB used in the current study could be accessed after the approval of the data application from Beijing Municipal Health Commission.

###  Measures

####  Catastrophic Health Expenditure 

 A household that incurs CHE was defined as the out-of-pocket health expenditures that exceed a certain threshold of a household’s capacity to pay.^4^ Out-of-pocket health expenditures were defined as the self-reported out-of-pocket total household expenditure on health-related services or products in the last year. Because household expenditure in HSSB is underestimated, this study used total household income to measure the capacity to pay of a household. A threshold of 40% was used to make our results comparable with previous studies in China.^15^


(1) $$CHE=\left\{\begin{array}{l} 1,if\frac{OOP}{CPH}\geq 40\%\\ 0,if\frac{OOP}{CPH}<40\% \end{array}\right.$$

where *CPH* is the capacity to pay of a household, and *OOP* is the out-of-pocket health expenditures.

####  Regional Factors

**Figure 1:**
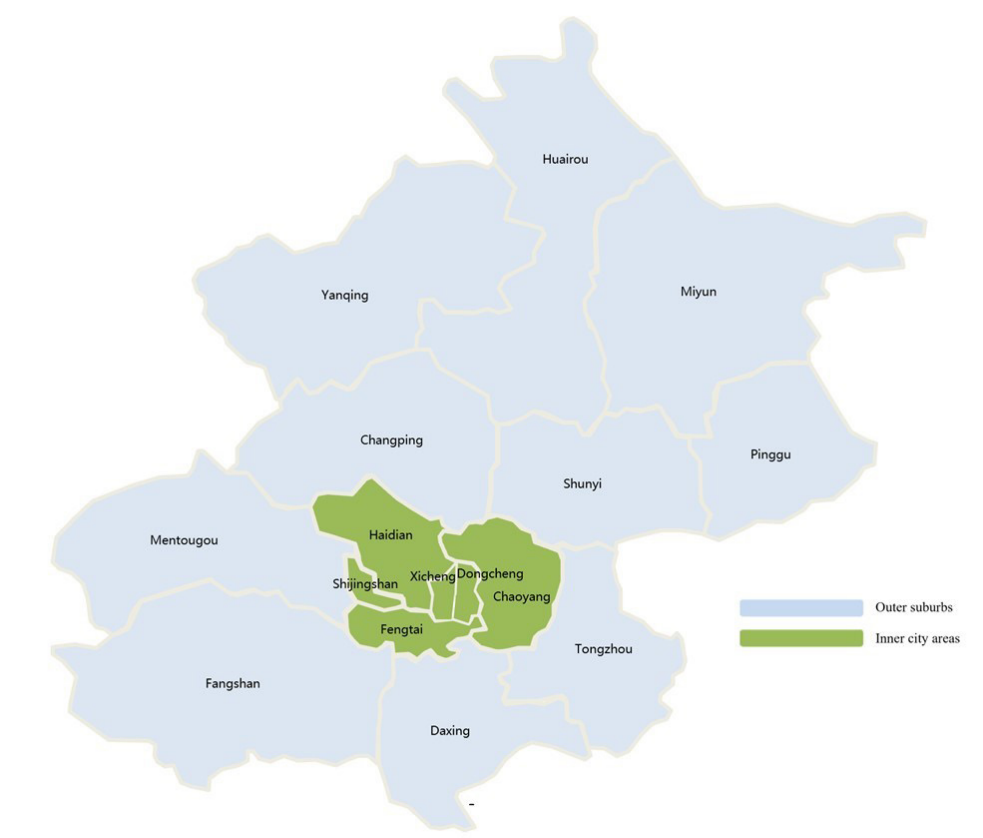
Map of Beijing.

 Regionally related factors were measured by the types of residential area at the community level. The types of residential areas were categorized as “villages in inner city areas,” “communities in inner city areas,” “villages in outer suburbs,” and “communities in outer suburbs” according to the classifications of residential regions in HSSB as villages or communities and the national standards for the division of 16 administrative districts (counties) of Beijing as inner-city areas and outer suburbs. Of these, villages and communities are both the smallest administrative units in China. The difference between the community and village is that the community is the administrative area in which the group within a fixed geographical area shares a common understanding and often the same language, manners, tradition and law, while the village is an administrative area with rural habitation. Figure 1 presents the map of Beijing presenting inner city areas and outer suburbs.

####  Compositional Factors

 We selected household income per capita, educational attainment, occupation, health insurance status, health insurance type and private health insurance status as the composition factors. Household income per capita was categorized as tertile 1 (the lowest household income per capita), tertile 2 or tertile 3 (the highest household income per capita). Educational attainment was based on the highest level attained by participants and was reclassified as primary school and below, junior high school, senior high school and college and above. Health insurance type was measured as URBMI, URRMI, other types of health insurance and the uninsured. Private health insurance status was classified as whether the individuals were not covered by private health insurance.

####  Covariates

 Covariates included age (continuous variable), gender (male or female), marital status (unmarried/married), self-reported health (good or bad), non-chronic diseases (yes or no), smoking (yes or no) and drinking (yes or no).

###  Statistical Analysis

 Descriptive analysis was used to describe the characteristics of the study sample and the prevalence of CHE. Logistic regression models were used to analyze the composition factors related to the risk of experiencing CHE of participants in different types of residential areas.


(2) $$logit\left(\frac{p}{1-p}\right)=\beta_{0}+\sum_{i=1}^{n}\beta_{i}x_{i}+\varepsilon$$

where* p* is the predicted probability that CHE is true for 1, *β*_0_. is the constant term, *β*_i_ is the odds ratio, *x*_i_ is the dependent variable, and *ε* is the error term.

 This study used concentration curves and the concentration index to estimate inequalities in CHE. Of these, concentration curves plotted the cumulative percentage of CHE against the cumulative percentage of the population ranked from poorest to richest (measured by the rank of household income per capita).^16^ The concentration index was used to analyze the income-related inequity of CHE, ranging from -1 to +1,^17^ which is calculated as:


(3) $$CI=\frac{2}{\mu }cov\left(y_{i}\text{,}r_{i}\right)$$

where *CI* is the concentration index, *μ* is the mean of the CHE indicator, *y*_i_ is the CHE indicator and *r*_i_ represents the fractional rank of households in the economic status distribution.

 After *CI* was calculated, a decomposition method based on a probit model was applied to quantify the contribution of each factor to income-related CHE inequity. A positive contribution indicates that the corresponding factor aggravates CHE inequality, and a negative contribution indicates a reduction in inequality.^18^ The decomposition method can be specified as follows:


(4) $$CI=\sum_{j}\left(\frac{\beta_{j}^{k}{\bar{x}}_{j}}{\mu }\right)C_{j}+\frac{GC_{\varepsilon }}{\mu }$$

where ${\bar{x}}_{j}$ is the mean of *x*_j_, $\beta_{j}^{k}$ is the partial effects (ie, d_CHE_/d_xj_) of each variable and evaluated at sample means, $\frac{\beta_{j}^{k}{\bar{x}}_{j}}{\mu }$ is the elasticity of *x*_j_ in CHE, *C*_j_ represents the concentration indices for *x*_j_, ε is the error term, and $\frac{GC_{\varepsilon }}{\mu }$ is the concentration index for the error term.

 A *P *value less than.05 was considered statistically significant. The software Stata version 15.0 for Windows (Stata Corp, College Station, TX, USA) was utilized for statistical analysis.

## Results

Table 1 shows the characteristics of the participants. A total of 37.74% of participants lived in communities of inner-city areas, and 6.87% resided in inner city areas’ villages; 39.19% of participants were from villages in outer suburbs, and 16.20% were from communities in outer suburbs. Among inner city areas residents, those living in villages were more likely to be more females, less in married, better health conditions, less smoking and drinking, lower educational attainment, unemployment or in low skill level occupation, with higher proportion of URRMI, less covered by private insurance than those living in communities. For participants in the outer suburbs, those with more females, better health conditions, less smoking and drinking, higher education level, high skill level occupation, more coverage by UEBMI and more private insurance tended to live in communities than in villages. More details can be found in Table 1.

**Table 1:** Characteristics of Participants, No. (%)/Mean (SD) (N = 25 297)

**Characteristics**	**Total (N = 25 297)**	**Inner City Areas**		**Outer Suburbs**	
		**Villages (n = 1737)**	**Communities (n = 9547)**	**Villages (n = 9915)**	**Communities (n = 4098)**
Income per capita					
Tertile 1 (lowest)	8476 (33.51)	574 (33.05)	3183 (33.34)	3250 (32.78)	1469 (35.85)
Tertile 2	7601 (30.05)	584 (33.62)	2751 (28.82)	2988 (30.14)	1278 (31.19)
Tertile 3 (highest)	9220 (36.45)	579 (33.33)	3613 (37.84)	3677 (37.09)	1351 (32.97)
Education					
Primary school and below	4271 (16.88)	272 (15.66)	781 (8.18)	2731 (27.54)	487 (11.88)
Junior high school	8225 (32.51)	715 (41.16)	1980 (20.74)	4455 (44.93)	1075 (26.23)
Senior high school	5721 (22.62)	444 (25.56)	2536 (26.56)	1801 (18.16)	940 (22.94)
College and above	7080 (27.99)	306 (17.62)	4250 (44.52)	928 (9.36)	1596 (38.95)
Occupation					
Unemployment	6351 (25.11)	243 (13.99)	464 (4.86)	4993 (50.36)	651 (15.89)
Skill level low	8440 (33.36)	916 (52.73)	3389 (35.50)	2895 (29.20)	1240 (30.26)
Skill level high	9308 (36.79)	494 (28.44)	5185 (54.31)	1637 (16.51)	1992 (48.61)
Student	1198 (4.74)	84 (4.84)	509 (5.33)	390 (3.93)	215 (5.25)
Insurance type					
UEBMI	13596 (53.75)	707 (40.70)	7193 (75.34)	3013 (30.39)	2683 (65.47)
URRMI	11001 (43.49)	1005 (57.86)	1962 (20.55)	6720 (67.78)	1314 (32.06)
Other	237 (0.94)	4 (0.23)	186 (1.95)	35 (0.35)	12 (0.29)
Uninsured	463 (1.83)	21 (1.21)	206 (2.16)	147 (1.48)	89 (2.17)
Private insurance	3319 (13.12)	221 (12.72)	1347 (14.11)	1165 (11.75)	586 (14.30)
Age, years, mean (SD)	51.60 (17.20)	50.12 (15.82)	51.28 (18.31)	52.78 (16.14)	50.14 (17.40)
Female	13103 (51.80)	890 (51.24)	5081 (53.22)	4966 (50.09)	2166 (52.86)
Married	20731 (81.95)	1465 (84.34)	7593 (79.53)	8241 (83.12)	3432 (83.75)
Self-reported health	23440 (92.66)	1612 (92.80)	9125 (95.58)	8794 (88.69)	3909 (95.39)
Nonchronic diseases	9136 (36.11)	634 (36.50)	3294 (34.50)	3783 (38.15)	1425 (34.77)
Smoking	5898 (23.32)	514 (29.59)	1733 (18.15)	2765 (27.89)	886 (21.62)
Drinking	5839 (23.08)	382 (21.99)	1718 (18.00)	2754 (27.78)	985 (24.04)

Abbreviations: UEBMI, urban employee basic medical insurance; URRMI, urban‒rural resident medical insurance; SD, standard deviation.

**Figure 2:**
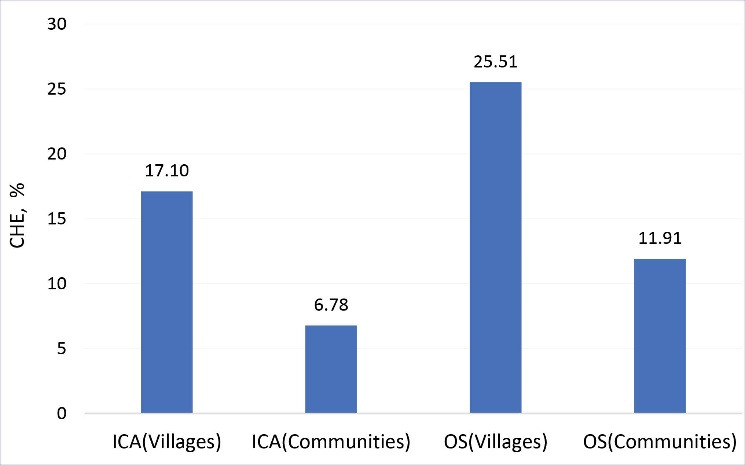
Prevalence of Catastrophic Health Expenditure by Types of Residential Areas. Abbreviations:ICA, inner-city areas; OS, outer suburbs; CHE, catastrophic health expenditure.

 The prevalence of CHE by types of residence and compositional factors is presented in Figure 2 and Table 2. Although villages in inner city areas had the highest out-of-pocket expenses (CNY 13 100) and villages in outer suburbs had the lowest out-of-pocket expenses (CNY 9443) (see Supplementary file 1, Table S1), villages of the outer suburbs experienced the highest prevalence of CHE (25.51%), and communities of inner-city areas had the lowest CHE prevalence (6.78%). A total of 17.10% and 11.91% of participants in villages of inner-city areas and communities of outer suburbs suffered from CHE, respectively. As the levels of income per capita, education and class of occupation increase, the range of CHE across different types of residential areas narrows. Compared with the regional CHE disparities in participants with uninsured health insurance, the disparities among those with UEBMI were smaller but larger in those with URRMI. Participants with private insurance coverage had smaller regional disparities in CHE than participants without private insurance coverage.

**Table 2:** Prevalence of Exposure to Catastrophic Health Expenditure, %

**Characteristics**	**Total**	**Inner City Areas**		**Outer Suburbs**		**Range**
		**Villages**	**Communities**	**Villages**	**Communities**	
Income per capita						
Tertile 1 (lowest)	28.14	28.92	10.71	47.38	23.01	36.67
Tertile 2	13.45	18.32	6.98	20.62	8.37	13.64
Tertile 3 (highest)	6.01	4.15	3.16	10.14	3.18	6.98
Education						
Primary school and below	30.95	23.16	12.55	37.61	27.52	25.06
Junior high school	18.44	18.04	10.30	22.96	14.98	12.66
Senior high school	11.82	16.22	6.27	18.27	12.34	12.00
College and above	6.30	10.78	4.38	16.16	4.82	11.78
Occupation						
Unemployment	31.55	23.05	12.93	34.37	26.42	21.44
Skill level low	13.57	17.90	9.06	17.58	13.31	8.52
Skill level high	7.35	12.96	4.96	14.17	6.58	9.21
Student	10.68	15.48	4.52	18.46	9.30	13.94
Insurance type						
UEBMI	9.33	13.15	6.03	16.96	8.57	10.93
URRMI	23.62	19.70	9.07	29.45	18.49	20.38
Other	8.86	50.00	8.60	8.57	0.00	8.57
Uninsured	15.98	19.05	9.22	24.49	16.85	15.27
Private insurance						
No	16.58	17.74	7.16	26.57	13.15	19.41
Yes	9.58	12.67	4.45	17.51	4.44	13.06

Abbreviations: UEBMI, urban employee basic medical insurance; URRMI, urban‒rural resident medical insurance.

 The association between compositional factors and CHE is presented in Table 3. A lower level of income contributed to a higher risk of CHE in the four types of residential areas. For example, among residents in villages of inner city areas, compared with the lowest tertile income group, the middle tertile income group and the highest tertile income group related to a decrease of 48% and 89% risk of CHE, respectively. Their community living counterparts were related to a 42% and 72% decrease in the risk of CHE in the middle tertile income group and the highest tertile income group, respectively. Although there was no significant association of education with CHE in inner city areas and outer suburbs’ villages, the college and above group in outer suburbs’ communities was more likely to have a lower risk of CHE than the group with primary school and below level of education. No significant association between occupation and CHE was found in inner city areas. Additionally, private insurance coverage was related to a decreased risk of CHE in residents of outer suburbs’ communities, with an odds ratio of 0.54 (95% confidence interval: 0.35-0.83).

**Table 3:** Association of Compositional Factors With Catastrophic Health Expenditure by Types of Residential Areas (N = 25 297)

**Characteristics**	**Inner City Areas ** **OR (95% CI)**		**Outer Suburbs ** **OR (95% CI)**	
	**Villages**	**Communities**	**Villages**	**Communities**
Income per capita				
Tertile 1 (lowest)	Reference	Reference	Reference	Reference
Tertile 2	0.52 (0.39, 0.70)^a^	0.58 (0.48, 0.70)^a^	0.32 (0.28, 0.36)^a^	0.31 (0.24, 0.40)^a^
Tertile 3 (highest)	0.11 (0.07, 0.17)^a^	0.28 (0.22, 0.35)^a^	0.15 (0.13, 0.17)^a^	0.14 (0.10, 0.21)^a^
Education				
Primary school and below	Reference	Reference	Reference	Reference
Junior high school	0.99 (0.67, 1.46)	1.12 (0.84, 1.49)	0.94 (0.82, 1.06)	0.75 (0.55, 1.01)
Senior high school	0.94 (0.60, 1.49)	0.85 (0.62, 1.15)	0.86 (0.72, 1.03)	0.80 (0.57, 1.13)
College and above	0.72 (0.40, 1.31)	0.99 (0.70, 1.39)	1.25 (0.97, 1.62)	0.61 (0.39, 0.95)^c^
Occupation				
Unemployment	Reference	Reference	Reference	Reference
Skill level low	0.95 (0.65, 1.38)	0.79 (0.56, 1.10)	0.75 (0.65, 0.87)^a^	0.73 (0.54, 0.99)^c^
Skill level high	0.98 (0.61, 1.56)	0.72 (0.51, 1.02)	0.76 (0.62, 0.94)^a^	0.72 (0.50, 1.02)
Student	0.60 (0.23, 1.54)	0.84 (0.46, 1.52)	1.33 (0.92, 1.94)	1.22 (0.59, 2.51)
Insurance type				
UEBMI	Reference	Reference	Reference	Reference
URRMI	1.21 (0.87, 1.70)	1.02 (0.82, 1.26)	0.92 (0.79, 1.06)	0.86 (0.66, 1.13)
Other	4.42 (0.55, 35.22)	1.54 (0.89, 2.68)	0.31 (0.09, 1.07)	-
Uninsured	1.07 (0.33, 3.51)	1.62 (0.97, 2.72)	0.96 (0.62, 1.49)	1.01 (0.53, 1.91)
Private insurance				
No	Reference	Reference	Reference	Reference
Yes	0.96 (0.61, 1.50)	1.07 (0.80, 1.43)	0.90 (0.76, 1.08)	0.54 (0.35, 0.83)^b^

Abbreviations: UEBMI, urban employee basic medical insurance; URRMI, urban‒rural resident medical insurance; CI, confidence interval; OR, odds ratio. All models adjusted for age, gendersex, marital status, self-reported health, nonchronic diseases, smoking and drinking. ^a^ *P *< .001, ^a^*P *< .005, ^c^*P *< .05.

**Figure 3:**
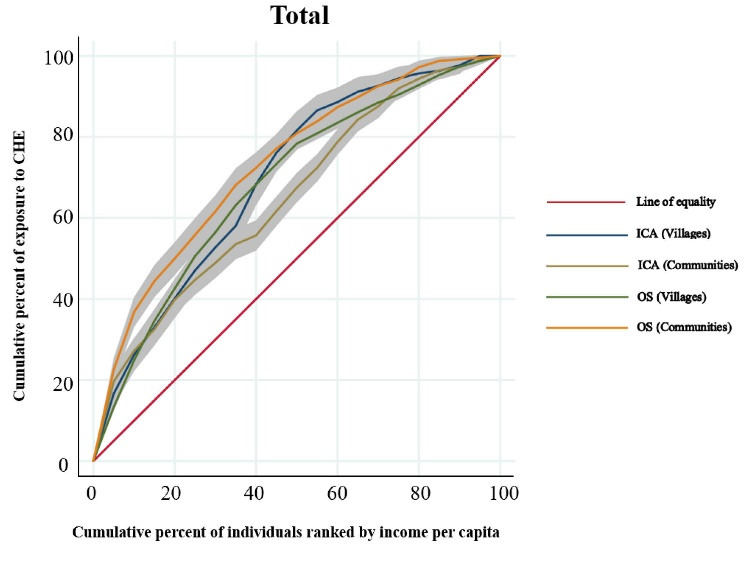
Concentrate Curve of Catastrophic Health Expenditure by Areas. Abbreviations:ICA, inner-city areas; OS, outer suburbs; CHE, catastrophic health expenditure.

 The slopes of the concentration curves in Figure 3 represent the inequalities of CHE in the four types of residential regions. Higher inequalities of CHE were in outer suburbs than in inner city areas. The concentration index analysis also supported this finding, with higher absolute values (-0.36) of concentration indices indexes in outer suburbs than in inner city areas (-0.13) (Supplementary file 1, Table S2). In the 0%-20% income group, higher rates of population with CHE were in the outer suburbs. In the 30%-50% income groups, the rates of people experiencing CHE in the outer suburbs continued to decrease. In the 50%-70% income group, CHEs continued a steady reduction among participants in the outer suburbs, while the upward trends were in inner city area residents. In the 70%-100% income group, the disparities in CHE inequalities between inner city areas and outer suburbs narrowed, and the rates of population with CHE decreased at similar rates. Additionally, the highest inequality of CHE was found in communities of the outer suburbs, which are mostly explained by higher levels of CHE in the lowest income groups and a reduction in CHE in the 30%-70% income group.

**Figure 4:**
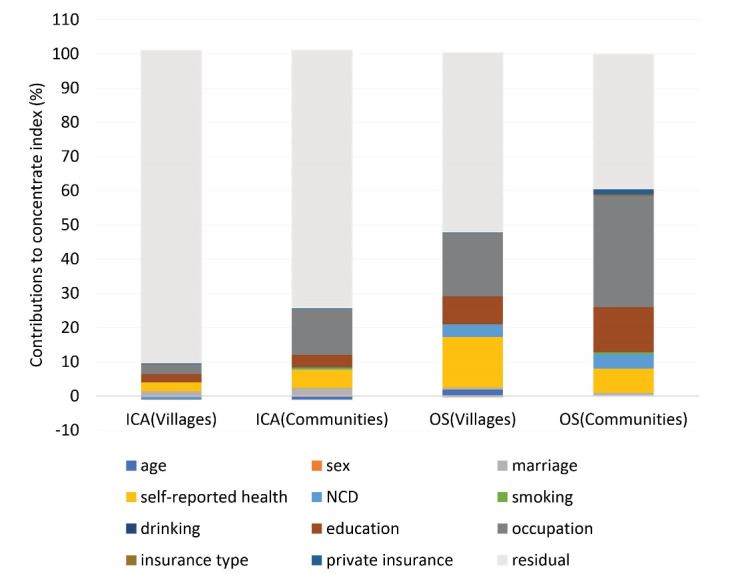
Decomposition of Inequality in Catastrophic Health Expenditure by Types of Residential Areas (N = 25 297). Abbreviations: ICA, inner-city areas; OS, outer suburbs; NCD, nonchronic disease.

Figure 4 presents the findings of the contributions of inequalities in CHE by four types of residential status. The total contribution percentage to CI was 8.73%, 24.78%, 47.67% and 60.58% for variables we involved among villages of inner-city areas, communities of inner city areas, villages of outer suburbs and communities of outer suburbs, respectively. The results indicated that 91.27%, 75.22%, 52.33% and 39.42% of the positive contribution to CHE inequity is explained by the residual in corresponding areas, respectively. Our findings showed that occupation and education are the two factors with the highest contributions of compositional factors to CHE inequalities.

## Discussion

 Identifying the occurrence of CHE and its regional inequity in megacities is essential for finding the existing barriers and effective ways of achieving the goals of UHC. Although several studies in China have focused on the occurrence of CHE and its income-related inequalities and presented similar findings (ie, inequalities in CHE associated with socioeconomic status [SES]),^19,20^ we know very little about regional-based inequalities of CHE in metropolitan areas. This study is the first to compare the incidence and inequality of CHE across different types of residential areas and to explore the contribution of compositional factors to the reduction of CHE and its inequality in Chinese megacities. Taking Beijing as an example, a large disparity in the incidence of CHE was found among the four types of residential areas. Generally, residents of villages in both outer suburbs and inner-city areas had a higher CHE incidence than residents of communities in the counterpart areas. Higher CHE inequalities were found in outer suburbs than in inner city areas.

 Our comparative analysis of CHE incidence in communities and villages located in inner city areas or outer suburbs showed that the highest incidence of CHE was in villages located in suburban areas, followed by villages from the urban cores. In Beijing, villages of suburban areas are commonly inhabited by rural residents with disadvantaged socioeconomic conditions and fewer financial protections from medical insurance schemes. Those residents are more likely to experience a high risk of diseases and unhealthy lifestyles (Table 1), which expands the health expenditures, and are more likely to experience CHE. In addition, villages located in urban cores of Beijing are also gathered around poor dwellers. These villages are often known as “urban villages,” inhabiting lower socioeconomic conditions, residents with squalor, overcrowding and social problems, and rural-to-urban migrants with smaller household sizes and less risk sharing among household members.^19,20^ In our analysis, only approximately 40% of residents in ‘urban villages’ were covered by UEBMI, and approximately 58% of them were covered by URRMI (with lower insurance coverage than UEMBI). Therefore, more concerns need to be diverted into the reduction of disease economic burden in households from both “urban villages” and villages in the outskirts of megacities.

 Strong pro-rich financing inequalities in CHE in Beijing were found in our results, especially in the outer suburbs. Although China’s universal coverage of basic health insurance and integration of URBMI and NCMS into the new URRMI scheme programs significantly increased healthcare utilization of inpatients and outpatients and the fairness in health insurance coverage,^21-23^ the subsidy from government actually benefits more the comparatively rich groups,^24^ which in turn may increase the risk of incurring CHE for the vulnerable groups.^22,24,25^ Specifically, in the outer suburbs of Beijing, the lack of higher-tier hospitals or higher-quality resources may increase the pro-rich inequalities in CHE. A previous study indicated that approximately 70% of hospitals were located in inner city areas with an area of 735 km^2^, and only 30% of hospitals were located in outer suburbs with an area of 16 073 km^2^.^7^ Most of the higher-tier hospitals and more cost-effective delivery or utilization of healthcare services are inside the urban cores.^7,8^ In our further inequality analysis across regions (Supplementary file 1, Table S2), the higher rate of residents with insufficient payment capacity and higher larger income-related inequalities (Supplementary file 1, Table S2) were in the outer suburbs, aggravating the inequalities in CHE in the outer suburbs.

 Our findings indicate that the risk and inequality of CHE are modifiable in the post-poverty elimination era, in which a high SES is the main contribution of compositional factors in reducing both the risk and inequality of CHE. SES was regarded as an important modifiable social determinant of health. As for the definition of the American Psychological Association, SES is the social standing or class of an individual or group and is often measured by the intersection of education, income, and occupation.^26^ Those with lower SES are more commonly uninsured and have more limited access to preventive, primary, and specialized care, which tend to have adverse health outcomes and poorer health status.^27^ The Commission on Social Determinants of Health of WHO pointed to tackling modifiable social determinants as an essential way to improve health equity.^28^ For instance, Quebec of Canadian provides premium waivers and government subsidies to individuals without job or earn less than US$ 12 000 a year, children under the age of 18 years and individuals over 65 years old. And in Singapore, low-income people receive more subsidies. All of the above examples to some extent compensate and narrow the inequalities across different SES individuals.

 Thus, reducing the occurrence of CHE and its inequality has promising prospects in the megacity of China. In addition, we found financial protection effects of private health insurance on decreasing the risk of CHE in the outer suburbs, which is similar to a previous study.^29^ Further policy development, such as targeting supplementary medical insurance in developing regions, needs to be the primary focus. For example, the Beijing Inclusive Medical Insurance Program, in which healthy applicants and individuals with preexisting conditions can be insured and have access to claims with a low premium rate but are more insurable, may be of great help to be out of pocket reduction, particularly in vulnerable areas.

 To our knowledge, this is the first study to focus on the regional inequalities of CHE in the setting of a typical megacity in China and to consider the financial risks from medical expenses in the post-poverty elimination era in China. We first found that preventing the risk and reducing the inequality of CHE was modifiable. However, several limitations should be noted. First, due to the restriction of survey data, the cost of transportation and accommodation related to ill health are not included in out-of-pocket expenditure, and using income instead of expenses to measure the household ability to pay may lead to the biased estimation of CHE. Second, not all contribution factors and some potential unobservable household characteristics were included in this study owing to data unavailability, such as mental health status, severities of illnesses and the health conditions of other family members. Therefore, the results should be interpreted with caution. Third, we are only able to rule out associations between contributed compositional factors and CHE rather than causations because of the cross-sectional analysis. Future studies should perform more causal analysis on this issue. Fourth, this study used survey data from 2018, and within this expansion of time, many situations have changed. Therefore, further research on updating the dataset can be performed in the future.

## Conclusion

 We found that large regional disparities in the incidence and pro-rich inequality in CHE occurred in a typical megacity of China. In the post-poverty elimination era, the impoverishment from medical expenses and CHE still needs to be noted. The modifiable determinants, SES, may be one of the effective interventions to reduce the risk and narrow the inequality of CHE in megacities. Addressing the socioeconomic inequalities of health systems should be considered in the road of achieving the UHC. Furthermore, the financial protection effects of inclusive supplementary medical insurance on decreasing the probability of CHE in the outer suburbs should be noted.Last but not least, a certain number of populations with no residence, even citizenship in the areas. They are invisible and neglected and not included in those survey data or studies, which are one of the important issues in megacities around the world and are part of the most vulnerable population. More attention should be given to them when UHC aims “no one left behind.”

## Acknowledgements

 We thank all participants and data investigators for this study.

## Ethical issues

 The study protocol was approved by the Chinese government and conducted in accordance with the legal framework of the Statistical Law of the People’s Republic (version 1996) of China. All participants provided informed consent to the government of China.

## Competing interests

 Authors declare that they have no competing interests.

## Authors’ contributions

 YL: study concept and design, drafting the manuscript, data analysis, interpretation and revision of article. ZS and DG: revision of article. PH: study concept and design and critical revision of article for important intellectual content. All authors gave final approval of the version to be published.

## Funding

 This work was supported by Major Project of the National Social Science Fund of China (21&ZD187).

## Supplementary files


Supplementary file 1 contains Tables S1 and S2.
