# Supplementary material for: Toward Universal Health Coverage: Regional Inequalities and Potential Solutions for Alleviating Catastrophic Health Expenditure in the Post-poverty Elimination Era of China
Source: Int J Health Policy Manag. 2023 Feb 20;12:7332. doi: 10.34172/ijhpm.2023.7332 (PMC10125131; doi:10.34172/ijhpm.2023.7332)
Supplement: Supplementary file 1 — contains Tables S1 and S2. [file ijhpm-12-7332-s001.pdf]

Article title: Toward Universal Health Coverage: Regional Inequalities and Potential Solutions for Alleviating Catastrophic Health Expenditure in the Post-poverty Elimination Era of China

Journal name: International Journal of Health Policy and Management (IJHPM)

Authors' information: Yanan Luo[1], Zhenyu Shi[2,3], Dan Guo[2,3], Ping He[3]*

[1]Department of Global Health, School of Public Health, Peking University, Beijing, China.

[2]School of Public Health, Peking University, Beijing, China.

[3]China Center for Health Development Studies, Peking University, Beijing, China.

(*Corresponding author: phe@pku.edu.cn)

Supplementary file 1

Table S1. Out-of-pocket expenses across the regions in Beijing, CNY, mean (SD)

Characteristic	Out-of-pocket expenses
**Inner city areas**	
Villages	13100 (20114)
Communities	12977(20415)
**Outer suburbs**	
Villages	9443(18261)
Communities	10847(15435)

Table S2. Gini Index, proportion of low-income group and CI across the regions in Beijing

Characteristic	Gini Index	Low-income group (%) [a]	CI
**Inner city areas**	**0.42**	**13.82**	**-0.13**
Villages	0.35	41.16	-0.27
Communities	0.41	8.85	-0.08
**Outer suburbs**	**0.44**	**52.65**	**-0.36**
Villages	0.41	66.52	-0.38
Communities	0.44	19.11	-0.22

Note: [a] participants with household income per capita lower than the 1st tertiles among all survey respondents. CI, concentration index.
